# Comparative efficacy of six types of scoliosis-specific exercises on adolescent idiopathic scoliosis: a systematic review and network meta-analysis

**DOI:** 10.1186/s12891-024-08223-1

**Published:** 2024-12-26

**Authors:** Zhenghui Wang, Wenpan Zhu, Guang Li, Xuejun Guo

**Affiliations:** Department of Rehabilitation, The First Affiliated Hospital of Xinxiang Medical University, Weihui, China

**Keywords:** Scoliosis, Exercise, Systematic review, Meta-analysis

## Abstract

**Background:**

Adolescent idiopathic scoliosis (AIS) stands as the predominant spinal deformity in adolescents, manifesting symptoms including back pain, functional limitations, cosmetic worries, and respiratory dysfunction. At present, six approaches of scoliosis-specific exercises are globally practiced, encompassing Schroth exercise, the Scientific Exercise Approach to Scoliosis (SEAS), the Dobomed, the side shift exercise, active self-correction, and the Functional Individual Therapy of Scoliosis (FITS). However, there is no systematic review and meta-analysis comparing the efficacy of these six types of scoliosis-specific exercises on adolescent idiopathic scoliosis.

**Objective:**

To evaluate and compare the efficacy of six types of scoliosis-specific exercises on spinal deformity and quality of life in AIS.

**Materials and methods:**

A systematic search was performed on PubMed, EMBASE, and the Cochrane Library from their inception to September 2023. Two independent auditors screened all studies according to predefined inclusion and exclusion criteria. Clinical trials were compiled to investigate the effects of six exercise interventions on spinal deformity and quality of life in AIS.

**Results:**

Twenty-four studies were included, with a sample size of 1069 subjects. After meta-analysis, it was shown that SEAS ranked first in reducing Cobb angles (SUCRA: 84.8%); active self-correction and Schroth significantly improved the angles of trunk rotation in AIS (SUCRA: 86.6% and SUCRA: 79.1%, respectively); active self-correction and Schroth showed significant improvements in quality of life (SUCRA: 76.6% and SUCRA: 76.0%, respectively).

**Conclusion:**

According to the current findings, active self-correction demonstrated superior short-term benefits compared to other exercise interventions in ameliorating spinal deformity and improving quality of life for adolescents with idiopathic scoliosis. Meanwhile, Schroth exhibited long-term effects in improving both spinal deformity and quality of life.

**Registration information:**

This review was registered on PROSPERO on June 20, 2023 (ID: CRD42023433152).

**Supplementary Information:**

The online version contains supplementary material available at 10.1186/s12891-024-08223-1.

## Introduction

Scoliosis is characterized by a spinal deformity involving lateral deviation, axial rotation, and abnormal sagittal curvature of the spine [1]. Adolescent idiopathic scoliosis (AIS), the most prevalent form of scoliosis, lacks a clear etiology and typically emerges in the general population between the ages of ten and skeletal maturity [2]. The prevalence of AIS is roughly 2–3% in children between 10 and 16 years of age [3]. Adolescent idiopathic scoliosis may result in functional limitations, pain, cosmetic worries, respiratory issues, and the potential for progressive worsening during growth. These potential risks negatively affect quality of life (QoL) [4]. The diagnosis of AIS relies on the Cobb angle, which is used to quantify the spinal curve between the upper and lower limits of the deformity in the coronal plane. The diagnostic criterion of AIS is the coronal curvature equal to or greater than 10 degrees as observed on a standing anteroposterior X-ray image [5]. As for categorizing the severity of scoliosis, a curve up to 25° is classified as mild scoliosis, a curve between 25° to 45° is considered moderate scoliosis, and a curve above 45° is categorized as severe scoliosis [6].

Treatment strategies for AIS encompass surgery and non-surgical interventions. While surgery is typically recommended for severe AIS curves exceeding 45°, authors in the Cochrane review found no evidence supporting the efficacy of surgical interventions compared to non-surgical approaches for AIS patients with severe curves exceeding 45° [7]. Orthotic management is commonly prescribed with the Cobb angle between 25° and 50°. Patients with mild scoliosis are recommended to undergo exercise therapy. Properly administered exercise therapy can potentially postpone or obviate the need for bracing in AIS patients with curves measuring less than 25° [8]. Exercise therapy includes generalized physiotherapy (Pilates, Tai chi, and core exercise) and scoliosis-specific exercises (SSEs) [9]. SSEs are defined as curve-specific and individualized exercise programs taught in a rehabilitation center with the therapeutic aim of reducing spinal deformity [6]. The management of SSEs adheres closely to medical and physical assessments. SSE consists of seven schools including the scientific exercise approach (SEAS), Schroth, DoboMed, side shift exercise, active self-correction by Monticone, Lyon approach, and functional individual therapy of Scoliosis approach (FITS). Notably, the Lyon approach is not considered a traditional SSE as it primarily focuses on the combination of bracing and physiotherapy [10, 11]. Presently, Schroth exercises have evolved into various branches, encompassing Schroth best practice, Schroth 3D treatment, Schroth general and, the Barcelona Scoliosis Physical Therapy School approach (BSPTS) [10–12]. Two previous studies have shown that the efficacy of SSE on spinal curves for AIS was superior to general exercise [10, 13]. However, the Cochrane review of 2012 suggested that a lack of high quality evidence recommending the use of SSE for AIS [14]. Furthermore, the most recent Cochrane review indicated that only one high-quality study has demonstrated the effectiveness of SSEs over general exercise [15]. As of now, there is a need to bolster the evidence supporting the effectiveness of scoliosis-specific exercises.

In the present study, we define Schroth, SEAS, DoboMed, side shift exercise, active self-correction, and FITS as SSE. According to the results of the three latest systematic reviews, there is insufficient evidence to indicate that SSE could improve spinal deformity and quality of life [16–18]. Nevertheless, two meta-analyses on Schroth interventions revealed significant reductions in the Cobb angle and improvements in quality of life among adolescents with idiopathic scoliosis [12, 19]. Unfortunately, up to now, there is a lack of evidence examining the effectiveness of the other five types of scoliosis-specific exercises (SEAS, DoboMed, side shift exercise, active self-correction, and FITS) on AIS. Additionally, no systematic review or meta-analysis has compared the efficacy of all six schools of scoliosis-specific exercises (Schroth, SEAS, DoboMed, side shift exercise, active self-correction, and FITS) on adolescent idiopathic scoliosis.

Therefore, the aim of the present meta-analysis was to evaluate and compare the efficacy of six types of scoliosis-specific exercises on adolescent idiopathic scoliosis in terms of spinal deformity and quality of life.

## Materials and methods

### Search strategies

The present authors searched three electronic databases (Pubmed, EMBASE, and the Cochrane Library) from their inception to September 2023. The searching strategy was grounded in the PICOS principle: (P) population: adolescents with idiopathic scoliosis; (I) intervention: SEAS, Schroth, side-shift, DoboMed, active self-correction, and FITS exercise training; (C) Comparator: control group with routine care, bracing, orthotic treatment, and general exercise; (O) Outcomes: spinal curves and QoL scores for adolescents with idiopathic scoliosis; (S) Study type: clinical trials. The details are shown in Table 1 (Pubmed is shown as an example).

**Table 1:** Search strategy on PubMed

#1	“Scoliosis“[MeSH]
#2	(((Scoliosis[MeSH Major Topic]) OR (scolioses[Title/Abstract])) OR (spinal deformity[Title/Abstract])) OR (spinal disease[Title/Abstract])
#3	(((((((((Schroth[Title/Abstract]) OR (Scientific Exercises Approach[Title/Abstract])) OR (SEAS[Title/Abstract])) OR (Barcelona Scoliosis Physical Therapy[Title/Abstract])) OR (BSPTS[Title/Abstract])) OR (DoboMed[Title/Abstract])) OR (Side-shift[Title/Abstract])) OR (Functional Individual Therapy of Scoliosis[Title/Abstract])) OR (FITS[Title/Abstract])) OR (active self-correction[Title/Abstract])
#4	((((((((((((((((exercise[MeSH Major Topic]) OR (exercise intervention[Title/Abstract])) OR (exercise training[Title/Abstract])) OR (training[Title/Abstract])) OR (physical training[Title/Abstract])) OR (physical exercise[Title/Abstract])) OR (sports training[Title/Abstract])) OR (nurse intervention[Title/Abstract])) OR (routine care[Title/Abstract])) OR (standard care[Title/Abstract])) OR (bracing[Title/Abstract])) OR (orthotic[Title/Abstract])) OR (conservative care[Title/Abstract])) OR (conservative treatment[Title/Abstract])) OR (core stabilization[Title/Abstract])) OR (scoliosis-specific exercises[Title/Abstract])) OR (physiotherapeutic scoliosis-specific exercises[Title/Abstract])
	((#2) AND (#3)) AND (#4)

### Inclusion criteria

(1) The inclusion criteria for the study were structured as follows: (1) the experimental group comprised individuals undergoing one of six distinct exercise modalities (SEAS, Schroth, active self-correction, side-shift regimen, DoboMed technique, or FITS) for the treatment of AIS; (2) the control group consisted of individuals receiving routine care, bracing, or other rehabilitative interventions; and (3) outcomes encompassing at least one of the following parameters: the Cobb angle (measured in degrees), angle of trunk rotation (ATR) (measured in degrees), or QoL assessment using the SRS-22 or SRS-23 questionnaire.

### Exclusion criteria

(1) The exclusion criteria for the study were as follows: (1) studies with incomplete or unreported data; (2) studies sourced from protocols, conferences, or correspondence; and (3) studies that did not utilize control groups, as well as animal studies, protocols, conference abstracts, or correspondence.

### Study selection

Literature management software Endnote X9 was used to screen and exclude the literature. Two researchers initially screened titles and removed duplication, review papers, conference papers, protocols, and correspondence. Subsequently, the researchers reviewed the abstracts of the literature to identify relevant studies. Finally, both researchers independently examined the full texts of the remaining literature to determine if they met the inclusion criteria. Any discrepancies were resolved through discussion between the two researchers. If consensus could not be reached, a third researcher would intervene to resolve the disagreement. Ultimately, studies that remained consistent across the researchers were included for analysis.

### Data extraction

The extracted data was recorded under the following headings: (1) author (2), publication year (3), country (4), sample size (5), mean age (6), details of exercise programs, and (7) outcome measures.

### Risk of bias in individual studies

Eligible studies were assessed for risk of bias (ROB) at the study level following the Cochrane Handbook version 5.1.0 toll. The assessment of ROB for eligible studies considered seven key items: (1) randomized sequence generation (2), treatment allocation concealment, blinding of (3) subjects and (4) personnel (5), incomplete outcome data (6), selective reporting and (7) other sources of bias. According to the number of high-ROB components, studies were divided into three levels of risk of bias: high risk (five or more components), moderate risk (three or four), and low risk (two or less) [20].

Researchers were required to disclose where the data had been deposited and provide the accession numbers for the datasets. Ethical approval was not necessary for this meta-analysis, as it constitutes secondary research.

### Data analysis

In studies where exercise is the intervention, all variables are continuous variables reflected by means and standard deviation (SD) [21]. Continuous variables in the study are expressed as mean difference (MD = absolute difference between the means of two groups, defined as the difference in means between the treatment and control groups and calculated using the same scale) or standardized mean difference (SMD = mean difference in outcome between groups/standard deviation of outcome between subjects, used to combine data when trials with different scales) with 95% interval. A randomized effects model was used in the present study.

Meta-analysis was performed using Stata software (version 15.1) and Markov chain Monte Carlo simulation chains in a Bayesian-based framework according to the PRISMA NMA instruction manual [22, 23]. The agreement between direct and in-direct comparisons was quantified and shown using the nodal method. Consistency testing, conducted via Stata software, was considered successful if the P-value>0.05 [24].

Stata software visualizes and generates network diagrams of different exercise interventions. Within these diagrams, nodes represent different exercise interventions and control conditions. The lines connecting the nodes represent direct head-to-head comparisons between interventions. The size of the nodes and the width of the lines are proportional to the number of studies included in the comparisons [25].

A P score reflects the intervention hierarchy. The P score is regarded as a frequentist equivalent to the surface under the cumulative ranking curve (SUCRA) and is used to measure the extent of certainty that one treatment is superior to others, averaged over all competing interventions. The P score ranges from 0 to 1, where 1 indicates the best intervention strategy with no uncertainty, while 0 suggests the worst intervention strategy with no uncertainty. Even though the P score or SUCRA is always expressed as the percentage of effectiveness of exercise interventions, scores should be interpreted cautiously unless clinical differences exist between interventions [26]. To avoid publication bias in NMA caused by small-scale studies, a network funnel plot was generated and inspected based on the symmetry criterion [27].

## Results

### Study identification and selection

**Fig. 1:**
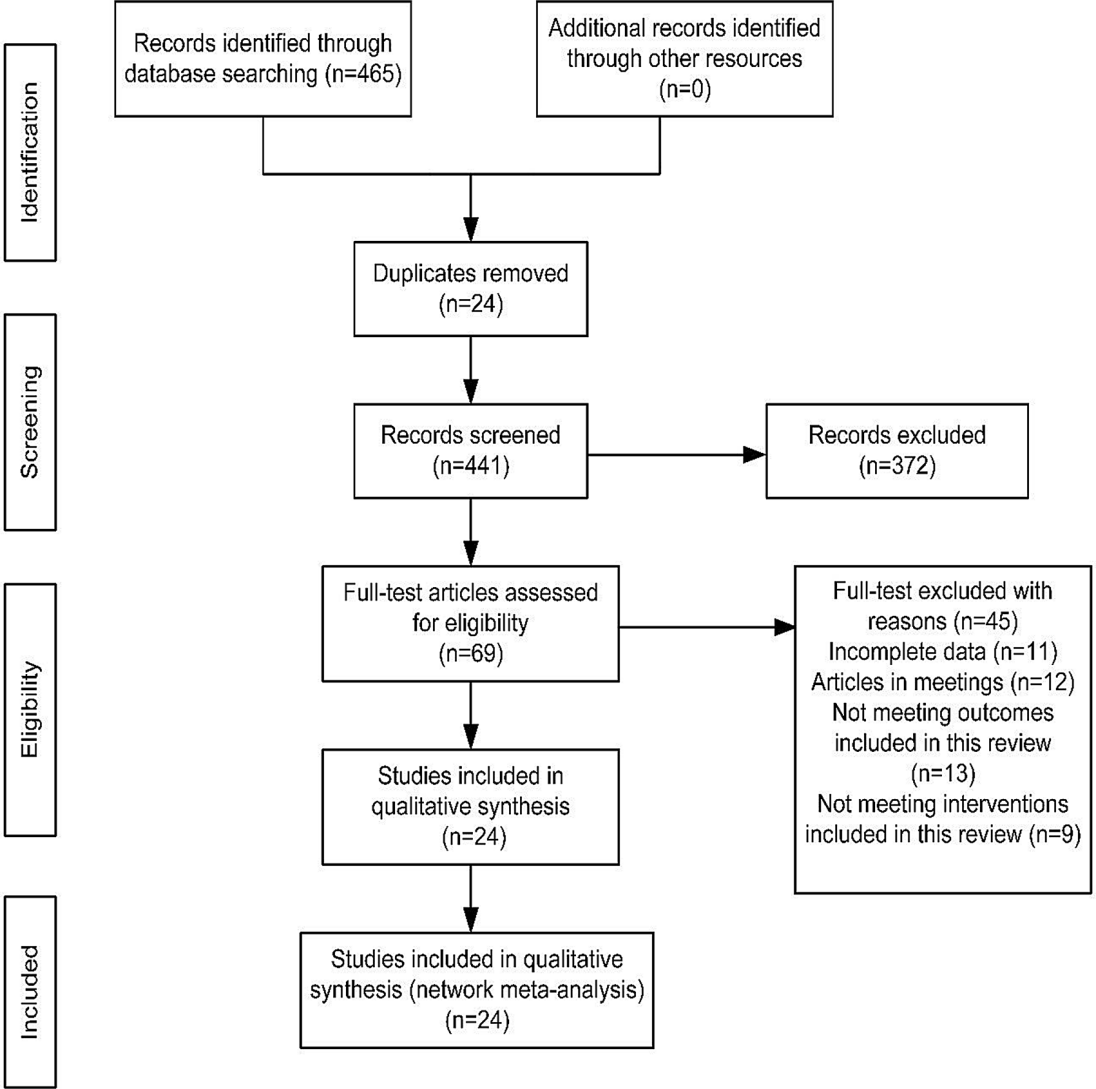
Flow diagram of literature selection. The flow of the search and selection process in this systematic review and meta-analysis of scoliosis-specific exercises

A total of 465 documents were searched from the electronic database. After removing duplicates, two researchers read the titles and abstracts of the remaining 441 documents, and then 372 documents were excluded. Moreover, full texts of the remaining 69 documents were read, and 45 documents were again excluded. Finally, 24 articles were left to be included in the present study. Specific details are presented in Fig. 1.

### Quality assessment of the included studies

**Fig. 2:**
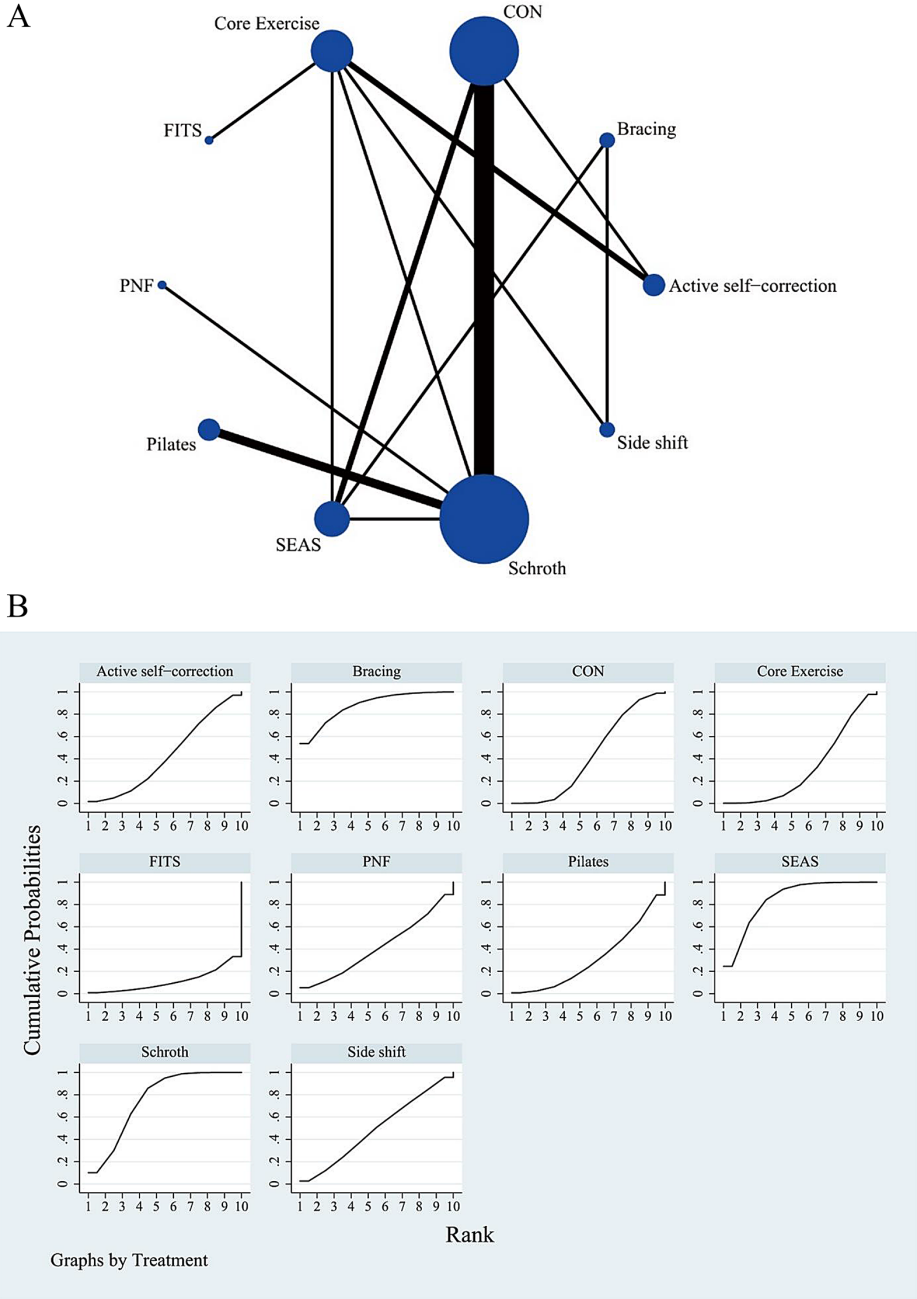
(**A**) Network meta-analysis map of intervention comparison for Cobb angle. Circle sizes represent the number of studies. Line widths indicate the number of direct comparisons. CON: control group with routine care (no exercise), SEAS: the Scientific Exercise Approach to Scoliosis, FITS: Functional Individual Therapy of Scoliosis, PNF: Proprioceptive Neuromuscular Facilitation. (**B**) Surface under the cumulative ranking curve plot for Cobb angle. The larger the surface, the larger the SUCRA, the better the efficacy of the intervention. CON: control group with routine care (no exercise), SEAS: the Scientific Exercise Approach to Scoliosis, FITS: Functional Individual Therapy of Scoliosis, PNF: Proprioceptive Neuromuscular Facilitation

Seventeen studies were classified as low risk, while four studies were categorized as moderate risk, and three studies as high risk. None of these studies implemented simultaneous blinding for participants and assessors, as participants or their relatives were required to provide informed consent prior to the exercise intervention. Specific details are presented in Supplementary Fig. 1 and Fig. 2 (refer to the appendix page).

### Characteristics of the included studies

In total, 24 studies were included, 17 of which were randomized controlled trials. The total sample size was 1069 patients with AIS. Intervention in the control group included routine care [28–38], bracing [39, 40], SEAS [41], and other modes of exercise programs such as Proprioceptive Neuromuscular Facilitation (PNF), Pilates, and core stabilization exercise training [10, 42–50]. Twenty-three studies reported the Cobb angle as an outcome indicator. Twelve studies reported ATR as an outcome, and nine recorded QoL scores as an outcome indicator. There were fourteen studies from Asia, five studies from Europe, three studies from North America, and two studies from Africa. The characteristics of each study are shown in Table 2.

### Network meta-analysis

**Fig. 3:**
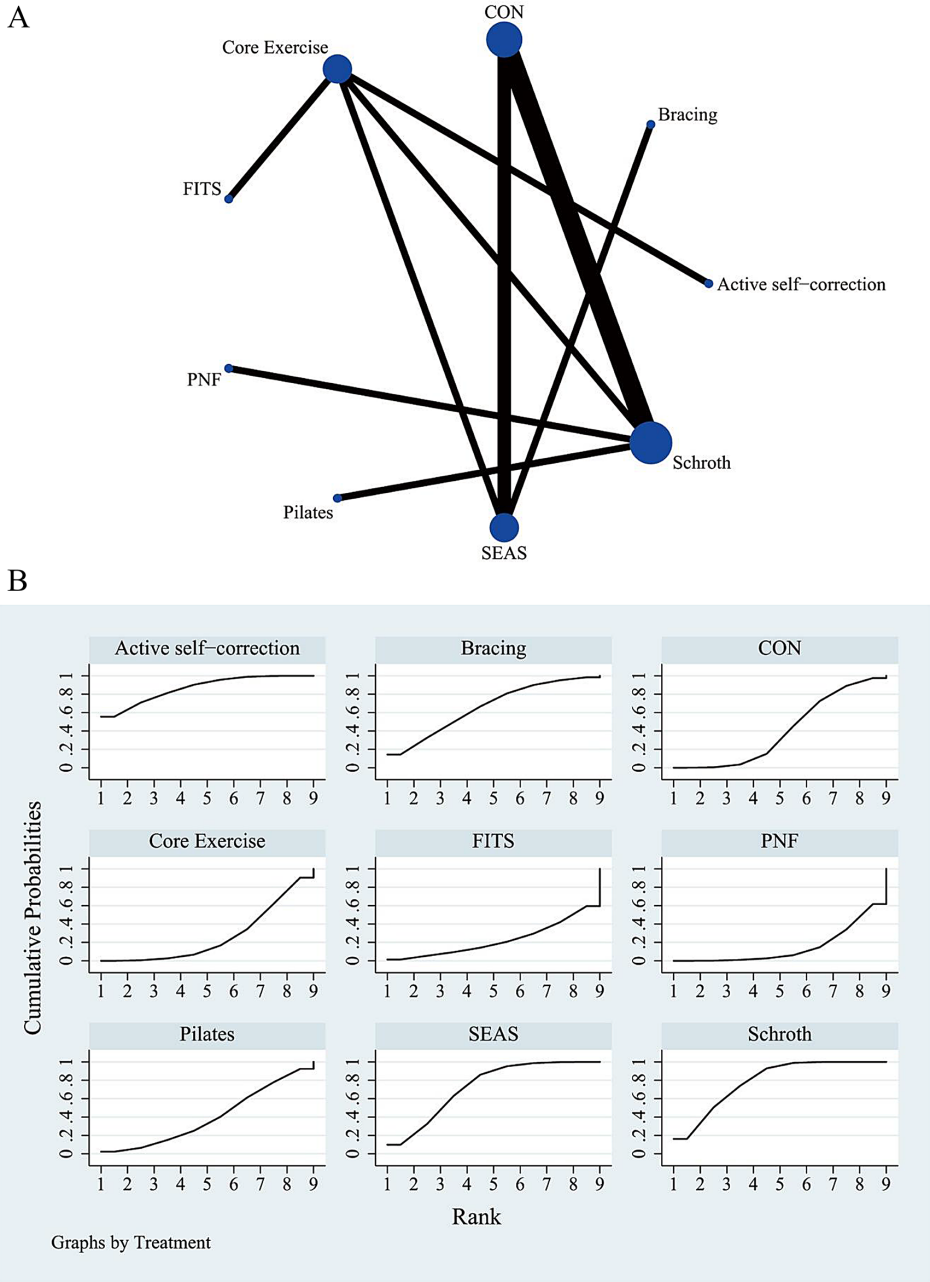
(**A**) Network meta-analysis map of intervention comparison for angle of trunk rotation. Circle sizes represent the number of studies. Line widths indicate the number of direct comparisons. CON: control group with routine care (no exercise), SEAS: the Scientific Exercise Approach to Scoliosis, PNF: Proprioceptive Neuromuscular Facilitation. (**B**) Surface under the cumulative ranking curve plot for angle of trunk rotation. The larger the surface, the larger the SUCRA, the better the efficacy of the intervention. CON: control group with routine care (no exercise), SEAS: the Scientific Exercise Approach to Scoliosis, PNF: Proprioceptive Neuromuscular Facilitation

**Fig. 4:**
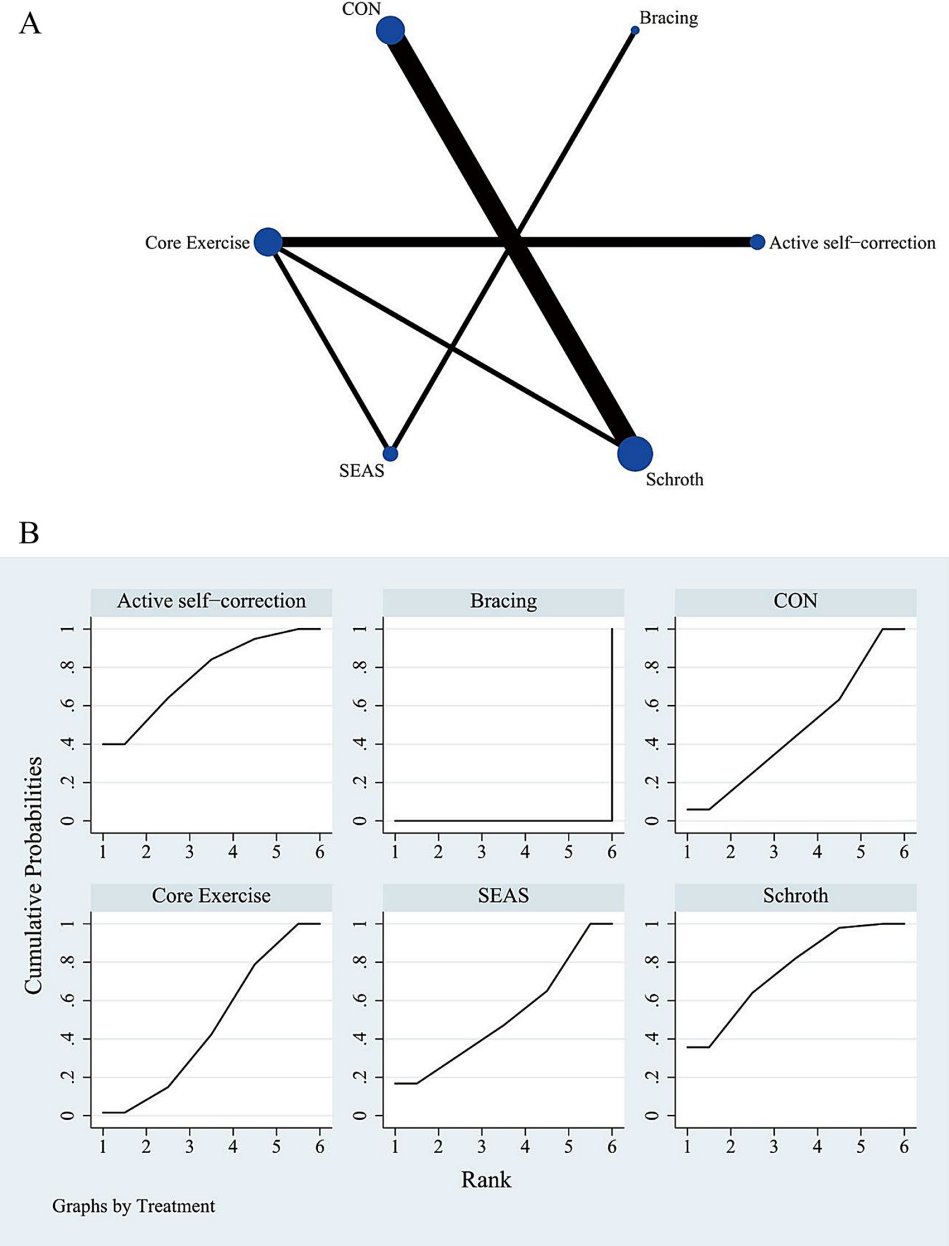
(**A**) Network meta-analysis map of intervention comparison for quality of life score. Circle sizes represent the number of studies. Line widths indicate the number of direct comparisons. CON: control group with routine care (no exercise), SEAS: the Scientific Exercise Approach to Scoliosis. (**B**) Surface under the cumulative ranking curve plot for quality of life score. The larger the surface, the larger the SUCRA, the better the efficacy of the intervention. CON: control group with routine care (no exercise), SEAS: the Scientific Exercise Approach to Scoliosis

The complete NMA figure is shown in Figs. 2A, 3A and 4A.

### Cobb angle

Consistency and inconsistency tests were conducted for direct and indirect comparisons, and all p-values were greater than 0.05. The result suggests that the consistency effects of all studies were acceptable. Additional details can be found in Supplementary Fig. 3 (refer to the appendix page).

Between the six types of scoliosis-specific exercises that were compared, the results of the Network meta-analysis showed that SEAS was superior to the control group (routine care) [MD=-4.09, 95%CI= (-8.00, -0.18)] and core exercise [MD=-5.24, 95%CI= (-10.28, -0.20)] in reducing the Cobb angles. Schroth was more effective than the control group (routine care) [MD=-2.86, 95%CI= (-5.63, -0.08)]. There were no significant differences in reducing Cobb angles between the other interventions. The probability of ranking different exercise interventions in reducing Cobb angles indicated that SEAS ranked first (SUCRA: 84.8%, as shown in Fig. 2B). A comparison between the two different interventions is presented in Table 3.

### Angle of trunk rotation

Indirect and direct comparisons between all studies were tested for consistency and inconsistency, and all P- values were greater than 0.05, eliciting that the consistency effect was acceptable. Additional details can be found in Supplementary Fig. 4 (refer to the appendix page).

The results of the meta-analysis suggest that active self-correction was superior to core exercise [MD=-3.00, 95%CI= (-4.77, -1.23)] and PNF [MD=-3.67, 95%CI= (-7.00, -0.33)] in the improvement of ATR. Schroth exercise was more effective in improving ATR than core exercise [MD=-2.27, 95%CI= (-4.48, -0.07)] and PNF [MD=-2.94, 95%CI= (-4.70, -1.18)]. Relative to PNF, SEAS was better in improving ATR [MD=-2.69, 95%CI= (-5.36, -0.02)]. Among the various exercise interventions, active self-correction ranked first in SUCRA for ATR improvement (SUCRA: 86.6%). Schroth secured the second position (SUCRA: 79.1%, as shown in Fig. 3B). A comparison between the two different interventions is depicted in Table 4.

### Quality of life score

Consistency and inconsistency tests were conducted for direct and indirect comparisons, and all p-values were greater than 0.05. The results suggest that the consistency effect between all studies was acceptable. Additional details can be found in Supplementary Fig. 5 (refer to the appendix page).

The results of the meta-analysis reveal that compared with bracing, active self-correction [MD = 3.46, 95%CI= (1.80, 5.11)], Schroth [MD = 3.43, 95%CI= (1.69, 5.17)], SEAS [MD = 3.17, 95%CI= (1.81, 4.53)], control group [MD = 3.15, 95%CI= (1.35, 4.95)], and core exercise [MD = 3.17, 95%CI= (1.60, 4.74)] were more effective in improving quality of life scores. The probability of ranking different exercise interventions showed that active self-correction ranked first (SUCRA: 76.6%). Schroth secured the second position in improving quality of life scores (SUCRA: 76.0%, as shown in Fig. 4B). A comparison between the two different interventions is presented in Table 5.

**Table 2:** Characteristics of the studies included in the meta-analysis

Author	Country	Year	Age(mean + sd)	Total/male/female	Intervention	Control	Outcome
Negrini, S.	Italy	2008	T:12.7(2.2) C:12.1(2.1)	T:35/10/25 C:39/12/27	SEAS exercise training Length of Intervention: 12 months Freq: 2 times a week Duration: 40 min Follow-up duration after treatment cession: NA	CON	Cobb angle, ATR
Zheng, Y.	China	2018	T:12.4(0.9) C:12.3(0.8)	T: 22/0/22 C: 19/0/19	SEAS exercise training Length of Intervention: 12 months Freq: 1 time a week Duration: 40 min Follow-up duration after treatment cession: 12 months	Bracing	Cobb angle, ATR, QoL score
Yagci, G.	Turkey	2019	T: 14.2(1.5) C: 14.0(1.3)	T: 15/0/15 C: 15/0/15	SEAS exercise training Length of Intervention: 4 months Freq: 1 time a week Duration: 40 min Follow-up duration after treatment cession: NA	Core stabilization exercises	Cobb angle, ATR, QoL score
Woo-Jin Lee	Korea	2017	T: 11(1.03) C: 10.07(1.00)	T: 14/9/5 C: 30/13/17	Side-shift exercise training Length of Intervention: 8 weeks Freq: 3 times a week Duration: NA Follow-up duration after treatment cession: NA	Core exercise training	Cobb angle
den Boer, W. A.	Netherlands	1999	T: 13.6(1.2) C: 13.6(1.3)	T: 44/NA/NA C: 120/NA/NA	Side-shift exercise training Length of Intervention: NA Freq: NA Duration: NA Follow-up duration after treatment cession: NA	Bracing	Cobb angle
Zapata, K. A.	USA	2019	T: 12.5(1.5) C: 11.8(0.9)	T: 19/7/12 C: 14/0/14	Schroth exercise training Length of Intervention: 6 months Freq: 3 times a week Duration: 15 min Follow-up duration after treatment cession: 6 months	CON	Cobb angle, QoL score
Kurak, K.	Turkey	2022	T: 15.50(1.68) C: 15.20(1.26)	T: 8/0/8 C: 8/0/8	Schroth exercise training Length of Intervention: 26 weeks Freq: 3 times a week Duration: 40 min Follow-up duration after treatment cession: NA	CON	Cobb angle, QoL score
Mohamed, R. A.	Egypt	2021	T: 14.50(1.20) C: 14.90(1.40)	T: 17/0/17 C: 17/0/17	Schroth exercise training Length of Intervention: 6 months Freq: 3 times a week Duration: 60 min Follow-up duration after treatment cession: 12 months	PNF training	Cobb angle, ATR
Kuru, T.	Turkey	2015	T: 12.9(1.4) C: 12.8(1.2)	T: 15/1/14 C: 15/2/13	Schroth exercise training Length of Intervention: 24 weeks Freq: 3 times a week Duration: 90 min Follow-up duration after treatment cession: NA	CON	Cobb angle, QoL score, ATR
Schreiber, S.	Canada	2016	T: 13.5(12.7–14.2) C: 13.3(12.7–13.9)	T: 23/NA/ NA C: 20/ NA/ NA	Schroth exercise training Length of Intervention: 6 months Freq: 7 times a week Duration: 30–45 min Follow-up duration after treatment cession: 6 months	CON	Cobb angle
Pil Neo HwangBo.	Korea	2018	T: 20.94 (0.32) C: 21.08 (1.95)	T: 8/0/8 C: 8/0/8	Schroth exercise training Length of Intervention: 12 weeks Freq: 3 times a week Duration: 60 min Follow-up duration after treatment cession: NA	Pilates	Cobb angle, ATR
Kocaman, H.	Turkey	2021	T: 14.07(2.37) C: 14.21(2.19)	T: 14/4/10 C: 14/3/11	Schroth exercise training Length of Intervention: 10 weeks Freq: 3 times a week Duration: 90 min Follow-up duration after treatment cession: NA	Core exercise training	Cobb angle, ATR, QoL score
Trzcińska, S.	Poland	2020	T: 13.53(1.33) C: 13.63(1.35)	T: 30/0/30 C: 30/0/30	FITS training Length of Intervention: 3 weeks Freq: 7 times a week Duration: 60 min Follow-up duration after treatment cession: NA	Core Exercise	Cobb angle, ATR
Gao, A.	China	2021	T: 15.1(1.6) C: 15.8(1.5)	T: 43/7/36 C: 21/4/17	Schroth exercise training Length of Intervention: 25–52 weeks Freq: 2–3 times a week Duration: 60 min Follow-up duration after treatment cession: 2 years	CON	Cobb angle, QoL score
Akyurek, E.	Turkey	2022	T: 13.73(1.83) C: 13.86(1.86)	T: 15/0/15 C: 14/0/14	Schroth exercise training Length of Intervention: 8 weeks Freq: 2 times a week Duration: 45–60 min Follow-up duration after treatment cession: NA	CON	ATR
Yuan, W.	China	2022	T: 8.0(7.0–9.0) C: 7.0(6.0–9.0)	T: 23/NA/NA C: 26/NA/NA	SEAS Length of Intervention: 1 year Freq: 5 times a week Duration: 30 min Follow-up duration after treatment cession: NA	CON	Cobb angle, ATR
Zapata, K. A.	USA	2023	T: 11.6(1.1) C: 12.5(1.4)	T: 34/NA/NA C: 19/NA/NA	Schroth exercise training Length of Intervention: 1 year Freq: 5 times a week Duration: 15 min Follow-up duration after treatment cession: NA	CON	Cobb angle
El Sayed Moubarak, E.	Egypt	2022	T: 12.03(1.94) C: 11.5(1.15)	T: 15/6/9 C: 15/5/10	Active self-correction Length of Intervention: 12 weeks Freq: 3 times a week Duration: 60 min Follow-up duration after treatment cession: NA	Core exercise training	Cobb angle, QoL score
Monticone, M.	Italy	2014	T: 12.5(1.1) C: 12.4(1.1)	T: 55/16/39 C: 55/14/41	Active self-correction Length of Intervention: 12 months Freq: 2 times a week Duration: 30 min Follow-up duration after treatment cession: NA	Core Exercise	Cobb angle, ATR, QoL score
Kumar A	India	2017	T: 11.56(1.46) C: 12.17(1.72)	T: 18/10/8 C: 18/11/7	Active self-correction Length of Intervention: 12 months Freq: NA Duration: 30 min Follow-up duration after treatment cession: NA	CON	Cobb angle
Kim G	Finland	2016	T: 15.60 (1.1) C: 15.3 (0.8)	T: 12/0/12 C: 12/0/12	Schroth exercise training Length of Intervention: 12 weeks Freq: 3 times a week Duration: 60 min Follow-up duration after treatment cession: NA	Pilates	Cobb angle
Pil Neo HwangBo.	Korea	2016	T: 18.14 (1.6) C: 18.88 (1.55)	T: 8/0/8 C: 8/0/8	Schroth exercise training Length of Intervention: 12 weeks Freq: 3 times a week Duration: NA Follow-up duration after treatment cession: NA	Pilates	Cobb angle
Lee, H-J	South Korea	2020	T: 18.88 (3.06) C: 24.14 (1.69)	T: 8/1/7 C: 7/1/6	Schroth exercise training Length of Intervention: 12 weeks Freq: 2 times a week Duration: 2 h Follow-up duration after treatment cession: NA	CON	Cobb angle, ATR
Shah J	India	2019	T: NA C: NA	T: 15/NA/NA C: 15/NA/NA	Schroth exercise training Length of Intervention: 7 weeks Freq: 5 times a week Duration: NA Follow-up duration after treatment cession: NA	SEAS	Cobb angle

*Note*: CON: control group with routine care (no exercise), T: experimental group, C: control group. SEAS: the Scientific Exercise Approach to Scoliosis, FITS: Functional Individual Therapy of Scoliosis, PNF: Proprioceptive Neuromuscular Facilitation. ATR: angle of trunk rotation, QoL: quality of life. Freq: frequency, NA: unavailable

**Table 3:**
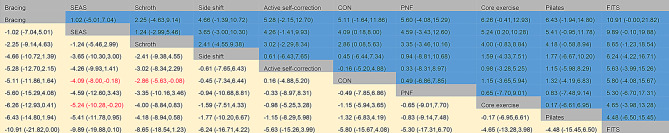
League table on Cobb angle

*Note*: CON: control group with routine care (no exercise), SEAS: the Scientific Exercise Approach to Scoliosis, BSPTS: the Barcelona Scoliosis Physical Therapy, FITS: Functional Individual Therapy of Scoliosis, PNF: Proprioceptive Neuromuscular Facilitation

**Table 4:**
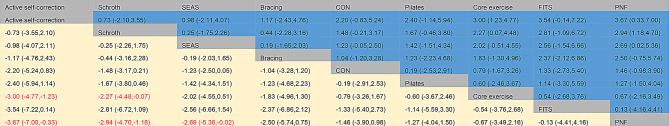
League table on ATR

*Note*: CON: control group with routine care (no exercise), SEAS: the Scientific Exercise Approach to Scoliosis, PNF: Proprioceptive Neuromuscular Facilitation

**Table 5:**
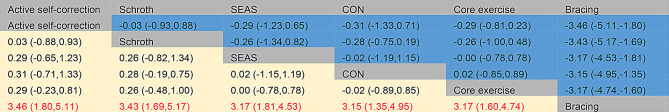
League table on QoL score

*Note*: CON: control group with routine care (no exercise), SEAS: the Scientific Exercise Approach to Scoliosis

### Publication bias test

Spare funnel plots of all outcome indicators were constructed to test possible publication bias. Visual inspection of the funnel plot did not indicate significant publication bias [51].

## Discussion

In the present meta-analysis, the effectiveness of five different scoliosis-specific exercises was compared to assess their impact on spinal deformity and QoL in adolescents with idiopathic scoliosis. As for the lack of DoboMed, no available clinical trials of DoboMed could meet the inclusion criteria. A total of 24 studies were included, including 1069 subjects diagnosed with AIS. The findings of the present study suggest that SEAS was the best SSE to reduce Cobb angles. However, active self-correction and Schroth exhibit better results in reducing ATR and improving HoL scores than other scoliosis-specific exercises. In summary, considering the improvement of spinal deformity and QoL, the present results indicate that active self-correction and Schroth are more effective than other types of scoliosis-specific exercises for AIS.

In the present review, five clinical trials of SEAS interventions were adopted, two of which [32, 34] were identified as high risk with low quality. Further, the sample size of these two studies accounted for more than 50% of the total number of subjects receiving SEAS intervention. Among all included studies, only one study [41] directly compared two types of SSE. This study suggested that Schroth was more efficacious than SEAS in reducing Cobb angles. Therefore, the conclusion that SEAS is the most effective exercise intervention in reducing Cobb angles should be interpreted with caution. Moreover, among these five trials, four [32, 34, 41, 48] did not report a follow-up period. It is crucial to incorporate a long follow-up period to gain insights into the long-term effects of exercise interventions. Due to the insufficient evidence available, it cannot be conclusively stated that SEAS has the ability to alter the natural progression of scoliosis. Overall, the lack of high-quality evidence limits the recommendation of SEAS as an effective strategy to reduce Cobb angles for AIS. Additionally, thirteen studies [28–30, 32, 34, 35, 37, 40–42, 44, 48, 49] were analyzed to assess the effect of Schroth on Cobb angles, with twelve of them [28, 29, 32, 34, 35, 37, 40–42, 44, 48, 49] indicating a significant difference in Cobb angles between the Schroth group and the control group. Among these twelve studies, the intervention duration for Schroth exercise lasted for a minimum of seven weeks. In line with Park JH’ recommendation, In line with Park JH’s recommendation, for better reduction of spinal deformity, AIS patients are advised to engage in Schroth exercise for at least four weeks [12]. Thus, long-term Schroth is more likely to have a better effect on Cobb angles’ reduction.

In terms of ATR, six clinical trials [10, 31, 42, 43, 45, 52] of active self-correction and Schroth were adopted in the present study, all of which were high-quality RCTs. Thus, the finding that active self-correction and Schroth may be the most effective types of scoliosis-specific exercises in ATP improvement is robust. The active self-correction program was formulated taking into account the specific attributes of the scoliotic curve. It aims to enhance spinal curvature, mobility, and strength, and address neuromuscular imbalances through targeted strategies such as strengthening deep spinal muscles, fostering postural self-correction, and incorporating stretches for the back muscles [10]. The core of Schroth exercise is to induce patients to de-collapse the concaved areas of trunks and reduce the prominences by using breathing mechanics, muscle activation, and mobilization. The implementation of Schroth exercise involves techniques such as rotational breathing and trunk stabilization training, which are aimed at decelerating and arresting the progression of ATR in AIS [53]. Further, four Schroth studies [29, 33, 36, 45] with a long follow-up duration showed that Schroth had a long-term influence on the ATR reduction. The studies on active self-correction did not mention a follow-up period. As such, the present results illustrate that active self-correction and Schroth have short-term effects on the ATR improvement for AIS. Additionally, Schroth exercise possesses a greater body of evidence supporting its ability to produce long-term improvements in ATR.

In the present study, three methods of SSE (active self-correction, Schroth, and SEAS) were adopted in investigating the effect of QoL improvement for adolescents with idiopathic scoliosis. The results show that active self-correction and Schroth had a better effect in improving QoL scores than other interventions. The ranking probabilities of these two intervention strategies were almost the same, SUCRA: 76.6% in active self-correction and SUCRA: 76.0% in Schroth, respectively. Previous meta-analyses [17, 19] utilizing RCTs have indicated that Schroth exercise significantly enhances QoL compared to the control group (routine care). Moreover, numerous studies have reported positive outcomes associated with active self-correction and Schroth exercises, including improvements in back muscle strength, breathing function, pain reduction, positive self-image, and a decrease in the prevalence of surgery [54–68]. These tangible effects theoretically improve patients’ QoL. However, due to the lack of follow-ups, the long-term effects of active self-correction on QoL improvement remain unclear.

## Strengths and limitations

Firstly, the present study compared the efficacy of improving spinal deformity and QoL between six types of scoliosis-specific exercises in AIS. By contrast, previous similar studies have merely focused on single scoliosis-specific exercises or the comparative efficacy between scoliosis-specific exercises and general exercises. Thus, novel and comprehensive evidence-based recommendations are provided in the present study.

Secondly, there are several limitations that influence the accuracy of the results. Heterogeneity caused by confounding factors between these original studies, such as the proportion of male or female participants, different regions, and scoliosis severity categories, was unavoidable. Finally, the results of the present study should be interpreted with caution because of the small sample size, with only 24 studies and 1069 participants. Except for Schroth, the number of studies investigating other scoliosis-specific exercises is extremely limited. Moreover, there is limited evidence from direct head-to-head comparisons between these six scoliosis-specific exercises. This underscores the need for additional relevant and high-quality studies to be conducted.

## Conclusions

Based on the present results, active self-correction demonstrates greater effectiveness than other exercise interventions in improving spinal deformity and quality of life for adolescents with idiopathic scoliosis in the short term. Additionally, Schroth exercise stands out for its effectiveness in both short-term and long-term improvements in spinal deformity and quality of life. Future studies should focus on evaluating the long-term outcomes of active self-correction exercise in adolescent idiopathic scoliosis.

## Electronic supplementary material

Below is the link to the electronic supplementary material.

Supplementary Material 1: Supplementary Fig. 1. Risk of bias summary.

Supplementary Material 2:Supplementary Fig. 2. Risk of bias graph.

Supplementary Material 3: Supplementary Fig. 3. Funnel plot of consistency effect for Cobb angles’ comparison between included studies.

Supplementary Material 4: Supplementary Fig. 4. Funnel plot of consistency effect for ATR comparison between included studies.

Supplementary Material 5: Supplementary Fig. 5. Funnel plot of consistency effect for QoL comparison between included studies.

## Data Availability

No datasets were generated or analysed during the current study.
