# Supplementary figures and images for: Comparative efficacy of six types of scoliosis-specific exercises on adolescent idiopathic scoliosis: a systematic review and network meta-analysis
Source: BMC Musculoskelet Disord. 2024 Dec 26;25:1070. doi: 10.1186/s12891-024-08223-1 (PMC11670383; doi:10.1186/s12891-024-08223-1)

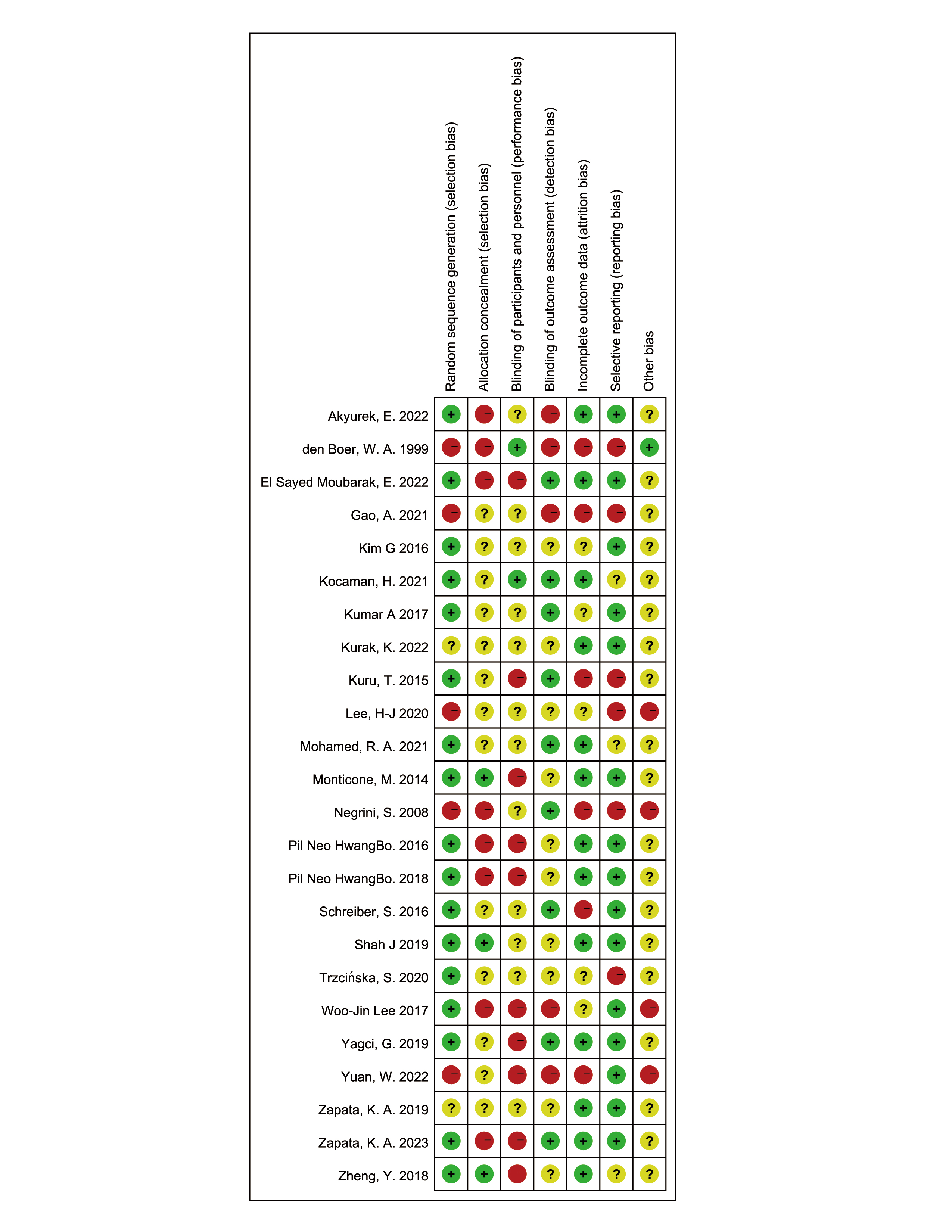

Supplement: Supplementary file 1 — Supplementary Material 1: Supplementary Fig. 1. Risk of bias summary. [file 12891_2024_8223_MOESM1_ESM.jpg]

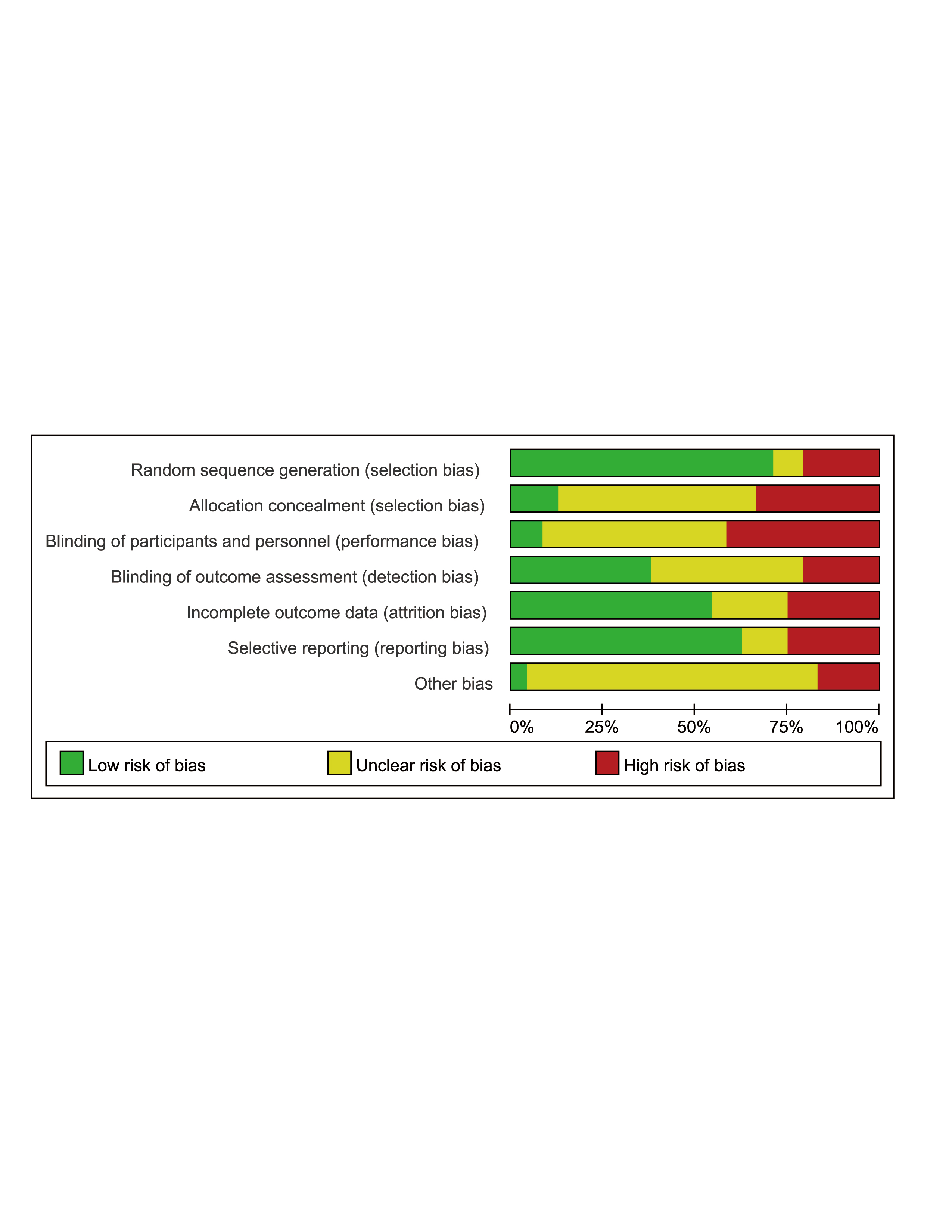

Supplement: Supplementary file 2 — Supplementary Material 2:Supplementary Fig. 2. Risk of bias graph. [file 12891_2024_8223_MOESM2_ESM.jpg]

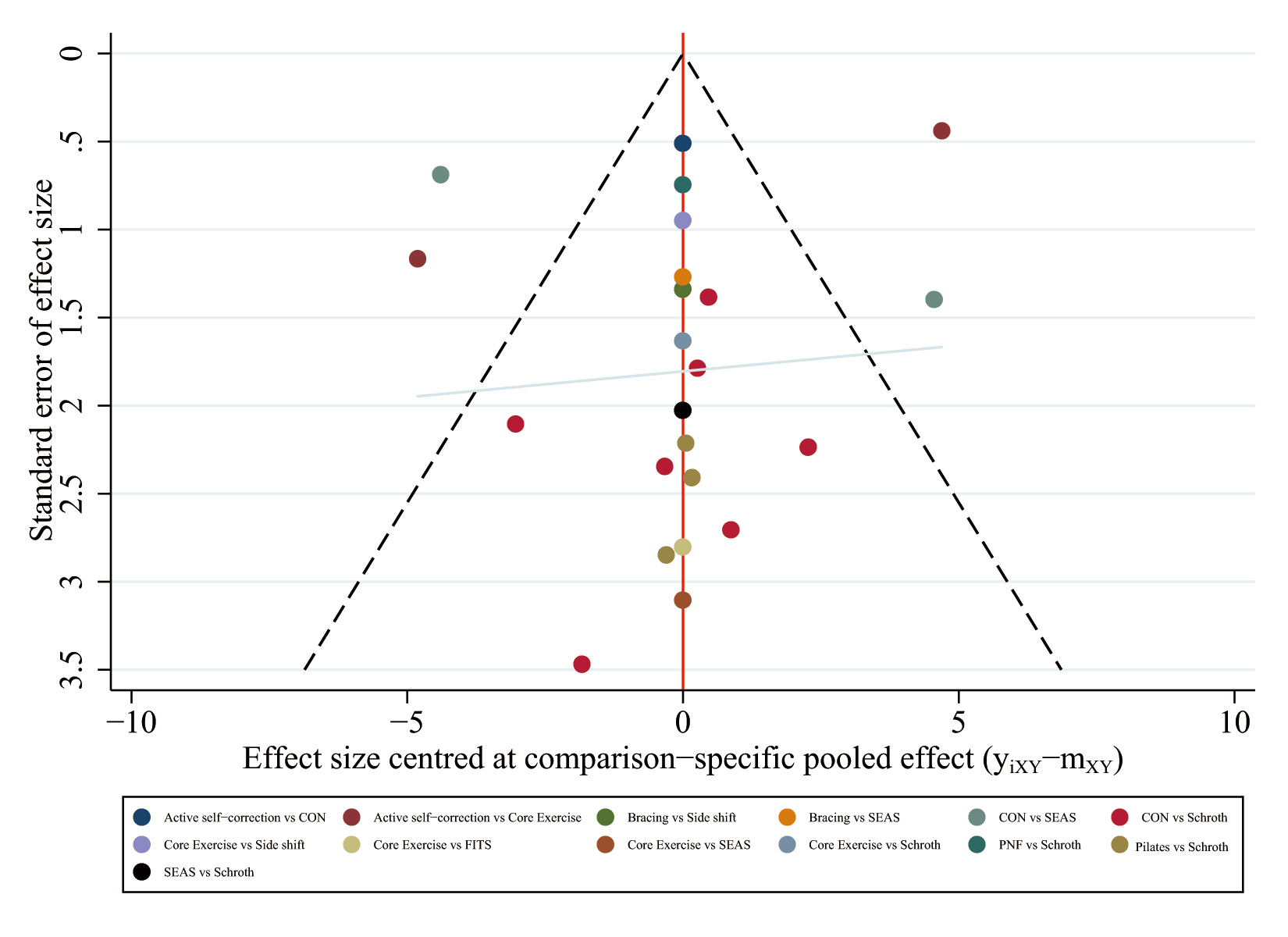

Supplement: Supplementary file 3 — Supplementary Material 3: Supplementary Fig. 3. Funnel plot of consistency effect for Cobb angles’ comparison between included studies. [file 12891_2024_8223_MOESM3_ESM.jpg]

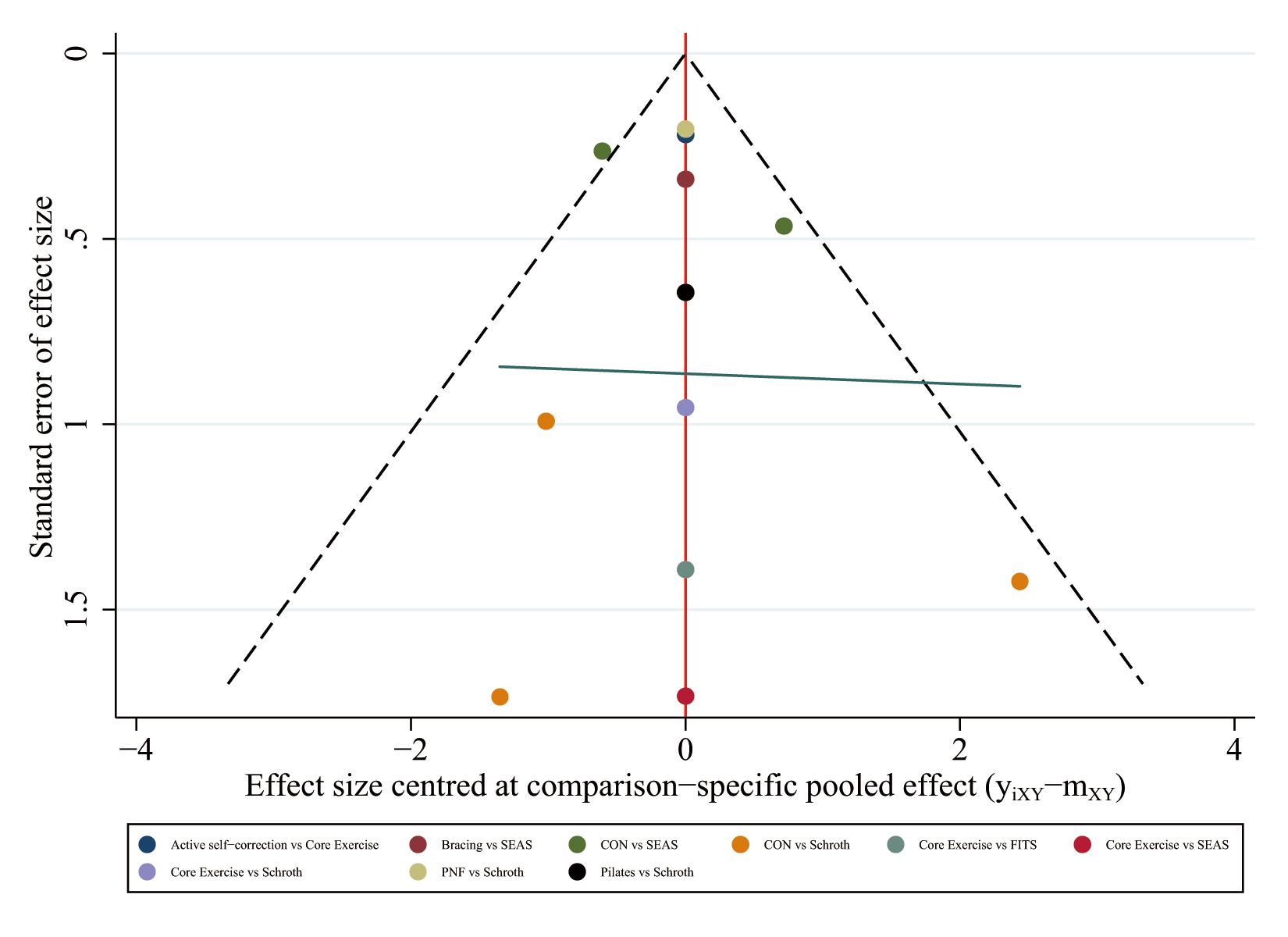

Supplement: Supplementary file 4 — Supplementary Material 4: Supplementary Fig. 4. Funnel plot of consistency effect for ATR comparison between included studies. [file 12891_2024_8223_MOESM4_ESM.jpg]

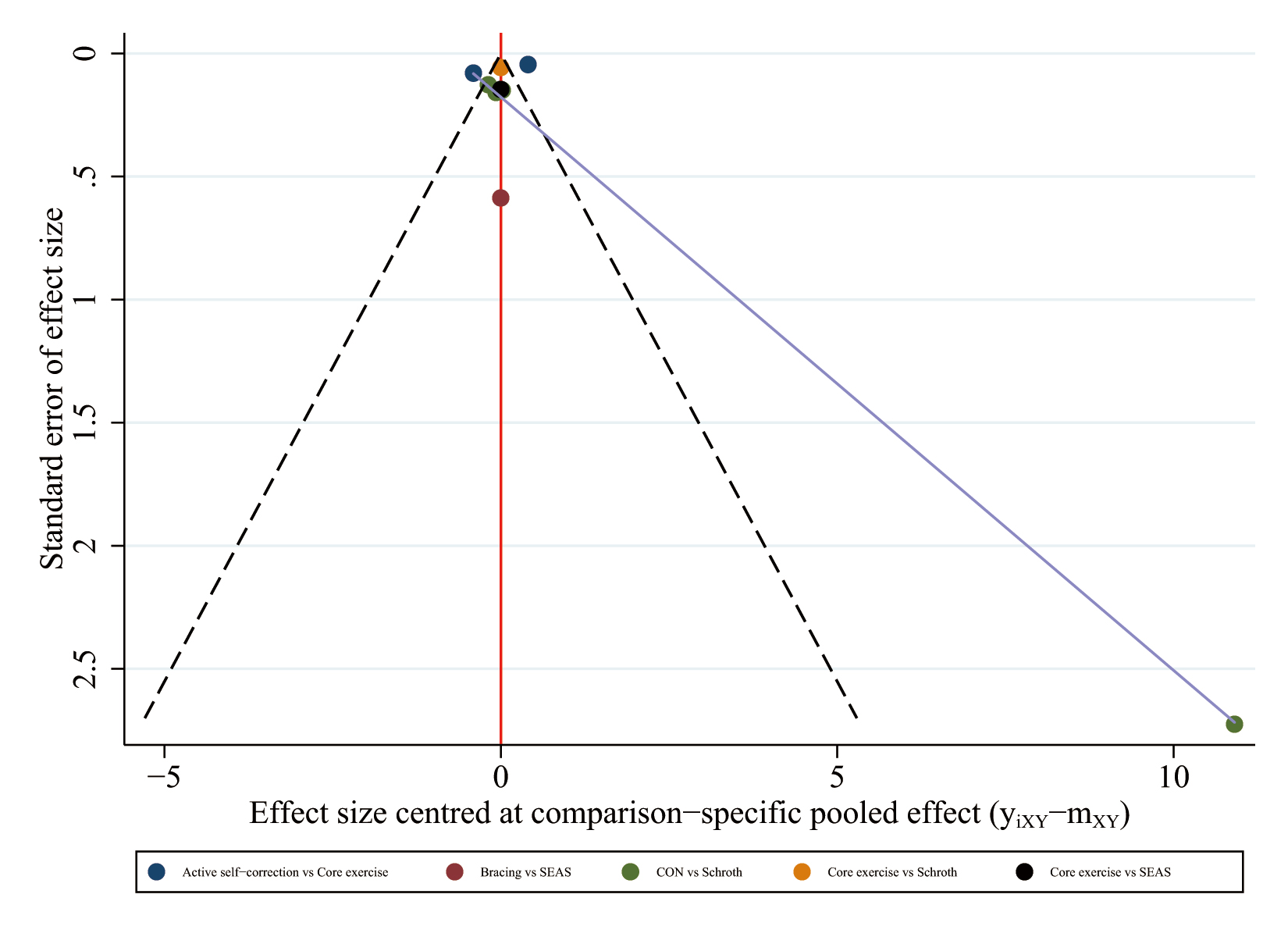

Supplement: Supplementary file 5 — Supplementary Material 5: Supplementary Fig. 5. Funnel plot of consistency effect for QoL comparison between included studies. [file 12891_2024_8223_MOESM5_ESM.jpg]
